# Treatment effect of *Bushen Huayu* extract on postmenopausal osteoporosis *in vivo*

**DOI:** 10.3892/etm.2014.1661

**Published:** 2014-04-02

**Authors:** LU OUYANG, QIUFANG ZHANG, XUZHI RUAN, YIBIN FENG, XUANBIN WANG

**Affiliations:** 1 Laboratory of Chinese Herbal Pharmacology, Renmin Hospital, Hubei University of Medicine, Shiyan, Hubei 442000, P.R. China; 2 School of Pharmacy, Hubei University of Medicine, Shiyan, Hubei 442000, P.R. China; 3 Basic School of Medicine, Hubei University of Medicine, Shiyan, Hubei 442000, P.R. China; 4 School of Chinese Medicine, LKS Faculty, The University of Hong Kong, Hong Kong, SAR, P.R. China

**Keywords:** traditional Chinese medicine, nilestriol, interleukin-6, alkaline phosphatase, estradiol

## Abstract

*Bushen Huayu* extract (BSHY), a traditional Chinese medicine, has been demonstrated to treat postmenopausal osteoporosis, however, the underlying mechanism remains to be fully elucidated. The aim of the present study was to investigate the therapeutic effect of BSHY and the mechanisms underlying this effect in an *in vivo* postmenopausal osteoporosis animal model. A total of 1 g BSHY containing 7.12 μg icariin was prepared. Low-dose BSHY (BSHY-L; 11.1 g/kg), medium-dose BSHY (BSHY-M; 22.2 g/kg) and high-dose BSHY (BSHY-H; 44.4 g/kg) was administered to oophorectomized rats using intragastric infusion. Estradiol (E2), interleukin-6 (IL-6) and serum alkaline phosphatase (ALP) levels, as well as bone density, were determined. It was found that the levels of serum ALP in the BSHY-L, BSHY-M and BSHY-H groups (197.75±41.74, 166.63±44.83 and 165.63±44.90 IU/l, respectively) were significantly decreased compared with the model group (299.13±45.79 IU/l; P<0.05), whilst the levels of E2 (16.89±1.71, 17.95±1.40 and 18.34±1.43 pg/ml, respectively) increased compared with the model group (14.54±1.61; P<0.05). In addition, the levels of IL-6 decreased in the BSHY-L, BSHY-M and BSHY-H groups (91.85±14.81, 82.99±15.65 and 80.54±14.61 pg/ml, respectively) compared with the model group (105.93±16.50 pg/ml; P<0.05). Furthermore, it was demonstrated that BSHY increased the bone density in the BSHY-L, BSHY-M and BSHY-H groups (0.20±0.014, 0.22±0.016 and 0.22±0.017 g/cm^2^, respectively) compared with the model group (0.19±0.011 g/cm^2^; P<0.05). BSHY was also found to increase the number of osteoblasts in the BSHY-L, BSHY-M and BSHY-H groups (25.38±2.17, 29.25±2.12 and 30.00±2.39, respectively), compared with in the model group (14.75±2.38; P<0.05), and decrease the number of osteoclasts in the BSHY-L, BSHY-M and BSHY-H groups (4.00±1.85, 4.25±1.39 and 5.75±1.49, respectively) compared with 9.50±1.60 observed in the model group (P<0.05). These results suggest that BSHY is a potential therapeutic drug for the treatment of osteoporosis *in vivo*. Furthermore, these results suggest that the mechanism by which BSHY decreases the serum levels of IL-6 may be by regulating E2.

## Introduction

Postmenopausal osteoporosis is a type of systemic bone disease, characterized by a reduction in bone density, degradation of bone microstructure and an alteration in serum markers of bone metabolism, including alkaline phosphatase (ALP), estradiol (E2) and interleukin-6 (IL-6) in postmenopausal females. This results in bone fragility and an increased risk of fracture. Approximately a third of postmenopausal females suffer from osteoporosis and this is usually due to the marked reduction in estrogen levels (1). Hormone replacement therapy (HRT) has been demonstrated to prevent bone loss and commonly includes a combination of estrogen, progesterone and progestin (2). However, long-term HRT results in adverse side effects, including hypocalcemia, worsening of renal impairment and osteonecrosis of the jaw (3), and therefore novel therapeutic strategies for the treatment of postmenopausal osteoporosis are required. *Bushen Huayu* (BSHY) is used in traditional Chinese medicine. It is based on the unique philosophy of Chinese medicine, namely *Bu Shen Hua Yu* (complementing the kidney system and resolving blood stasis). According to the theory of traditional Chinese medicine, BSHY is able to improve the function of the kidney system, strengthen bones, improve blood circulation and relieve pain (4). The aim of the present study was to investigate the therapeutic effects of BSHY on bone density and morphology, as well as on serum markers of bone metabolism in a postmenopausal osteoporosis animal model, and to investigate the underlying mechanisms.

## Materials and methods

### Drugs and reagents

All Chinese medicinal materials used in the present study were purchased from Hubei Shennong Bencao Prepared Herbal Medicines Co., Ltd. (Xuchang, Henan, China) and were identified by our laboratory. The herbarium specimens (no. SYRM-20090818-0090833) were deposited in Hubei University of Medicine (Shiyan, Hubei, China). The components were concentrated by decocting the solution twice in a 10-fold volume of water at 100°C for 30 min. It was found that 1 g BSHY corresponded with 1.2 g raw medicinal material. The concentration of icariin, a quantity reference compound in the extract, was 7.12 μg/g in a previous study (5). Nilestriol tablets were purchased from Shanghai Hualian Pharmaceutical Co., Ltd. (Shanghai, China; no. 970710). Chloral hydrate (10%) was freshly prepared in the laboratory. The E2 kit was purchased from Shenzhen Laerwen Bioengineering Technology Co., Ltd. (Shenzhen, Guangdong, China) and the IL-6 kit was purchased from Tianjin Jiuding Medical Bioengineering Co., Ltd. (Tianjin, China).

### Experimental animals

A total of 48 healthy, female Sprague-Dawley rats of SPF grade, aged 3 months and weighing 215±45 g were provided by the Hubei Laboratory Animal Center (Wuhan, Hubei, China; License no. SCXK-E2005-0008). The rats were randomly divided into six groups: the control group, the model group, the nilestriol group, the low-dose BSHY (BSHY-L) group, the medium-dose BSHY (BSHY-M) group and the high-dose BSHY (BSHY-H) group. All experiments were conducted in accordance with the Guidelines for Ethical Conduct in the Care and Use of Experimental Animals of the Hubei University of Medicine and were approved by the ethics committee of the university.

### Oophorectomy model establishment

The animal models were established as previously described with minor modifications (6). In the control group, sham surgery was performed and only a small piece of fat tissue near the ovary was removed. In all other groups, the ovaries were removed. Two weeks after the surgery, 11.1, 22.2 and 44.4 g/kg BSHY was administered by intragastric infusion to rats in the BSHY-L, BSHY-M and BSHY-H treatment groups, respectively, whilst nilestriol (0.1 mg/kg) was administered to rats in the nilestriol group. The animals in the control and model groups underwent intragastric infusions of 10 ml/kg of 0.9% sodium chloride (equal volume to the other groups). Treatment was performed for 12 weeks and was completed 24 h prior to sample collection.

### Sampling and detection

Following the drug treatment, blood and serum samples were collected from all the rats. Serum ALP, E2 and IL-6 levels were determined. The rats were sacrificed by cervical dislocation and the left and right femurs were collected. Dual energy X-ray absorptiometry was used to measure the bone mineral density in the right femur. Serial 4-μm sections were cut at the proximal end of the left femur and the slices were stained using hematoxylin and eosin. The slices were examined using microscopy (Olympus BX43, Olympus Co., Tokyo, Japan) and the average number of osteoblasts and osteoclasts was determined.

### Statistical analysis

All data are expressed as the mean ± standard deviation. The SPSS 13.0 software package was used for statistical analysis (SPSS, Inc., Chicago, IL, USA). Statistical differences were evaluated using one way analysis of variance. P<0.05 was considered to indicate a statistically significant difference.

## Results

### Effect of BSHY on the serum levels of E2 in oophorectomized rats

**Figure 1:**
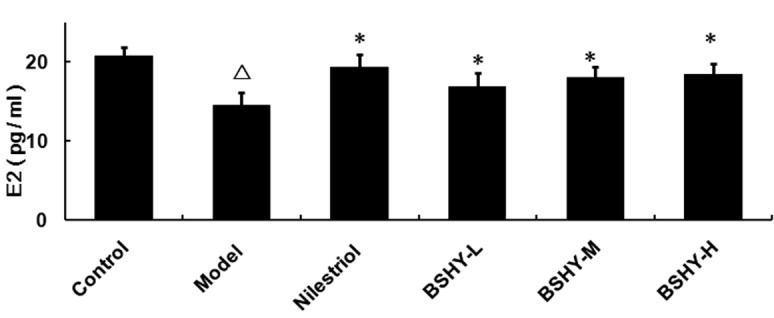
Effect of BSHY on serum E2 levels in rats. ^Δ^P<0.05, vs. the control group; ^*^P<0.05, vs. the model group. BSHY, *Bushen Huayu* extract; E2, estradiol; BSHY-L, low-dose BSHY; BSHY-M, medium-dose BSHY; BSHY-H, high-dose BSHY.

The serum E2 levels in the normal, model, nilestriol, BSHY-L, BSHY-M and BSHY-H groups were 20.71±1.08, 14.54±1.61, 19.34±1.59, 16.89±1.71, 17.95±1.40 and 18.34±1.43 pg/ml, respectively. The serum levels of E2 in the model group were significantly lower compared with the control group (P<0.05). The levels of E2 in the nilestriol group were significantly higher compared with the model group (P<0.05). Furthermore, compared with the model group, the serum levels of E2 in all three BSHY groups were significantly elevated (P<0.05), however, the levels remained lower than the control group (Fig. 1).

### Effect of BSHY on serum IL-6 levels in oophorectomized rats

**Figure 2:**
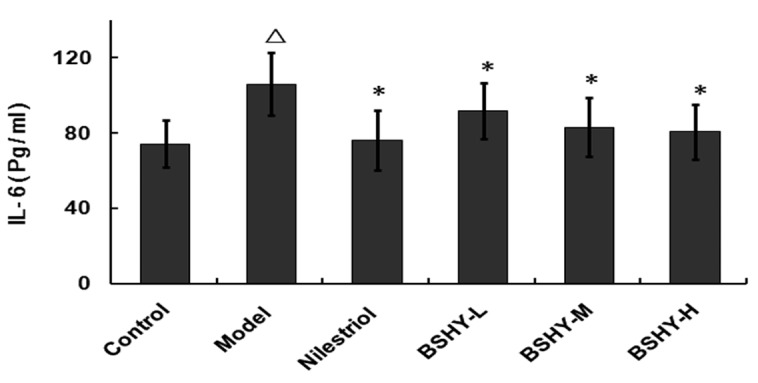
Effect of BSHY on serum IL-6 levels in rats. ^Δ^P<0.05, vs. the control group; ^*^P<0.05, vs. the model group. BSHY, *Bushen Huayu* extract; IL-6, interleukin-6; BSHY-L, low-dose BSHY; BSHY-M, medium-dose BSHY; BSHY-H, high-dose BSHY.

The serum levels of IL-6 in the normal, model, nilestriol, BSHY-L, BSHY-M and BSHY-H groups were 74.2±12.48, 105.93±16.50, 76.08±15.79, 91.85±14.81, 82.99±15.65 and 80.54±14.61 pg/ml, respectively. Compared with the control group, the serum levels of IL-6 in the model group were significantly increased (P<0.05), while the serum IL-6 levels in the BSHY-L, BSHY-M and BSHY-H groups were significantly lower compared with the model group (P<0.05; Fig. 2).

### Effect of BSHY on the serum levels of ALP in oophorectomized rats

**Figure 3:**
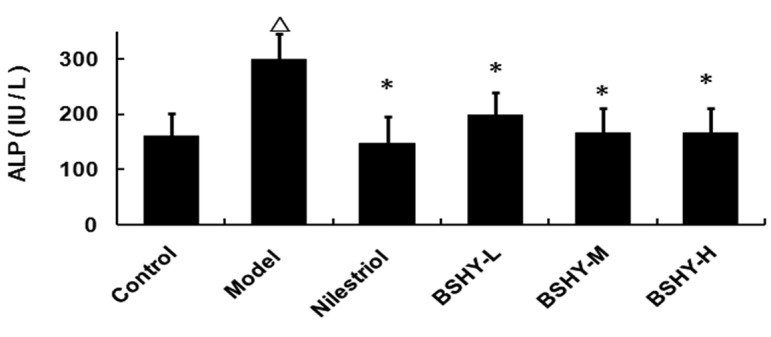
Effect of BSHY on serum ALP levels in rats. ^Δ^P<0.05, vs. the control group; ^*^P<0.05, vs. the model group. BSHY, *Bushen Huayu* extract; ALP, alkaline phosphatase; BSHY-L, low-dose BSHY; BSHY-M, medium-dose BSHY; BSHY-H, high-dose BSHY.

The serum ALP levels in rats in the normal, model, nilestriol, BSHY-L, BSHY-M and BSHY-H groups were 159.88±40.44, 299.13±45.79, 147.88±48.14, 197.75±41.74, 166.63±44.83 and 165.63±44.90 IU/l, respectively. Compared with the control group, the serum ALP levels of rats in the model group were significantly elevated (P<0.05), while the levels of ALP in the three BSHY treatment groups were significantly lower compared with the model group (P<0.05). However, no significant differences between the three BSHY groups and the nilestriol group were identified (P>0.05; Fig. 3).

### Effect of BSHY on bone density in the bone metaphysis of the rat right femur

**Figure 4:**
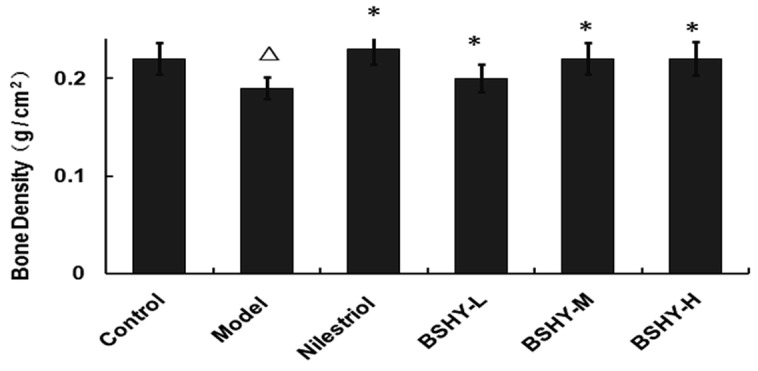
Effect of BSHY on bone density in the bone metaphysis of the rat right femur. ^Δ^P<0.05, vs. the control group; ^*^P<0.05, vs. the model group. BSHY, *Bushen Huayu* extract. BSHY-L, low-dose BSHY; BSHY-M, medium-dose BSHY; BSHY-H, high-dose BSHY.

The bone density in the metaphysis of the right femur in the normal, model, nilestriol, BSHY-L, BSHY-M and BSHY-H groups were 0.22±0.016, 0.19±0.011, 0.23±0.016, 0.20±0.014, 0.22±0.016 and 0.22±0.017 g/cm^2^, respectively. The bone density in the BSHY-L, BSHY-M and BSHY-H groups was elevated compared with the model group (P<0.05) and no significant differences between the BSHY treatment groups were observed (P>0.05; Fig. 4).

### Alterations in bone tissue morphology in the proximal end of the left femurs of oophorectomized rats

**Figure 5:**
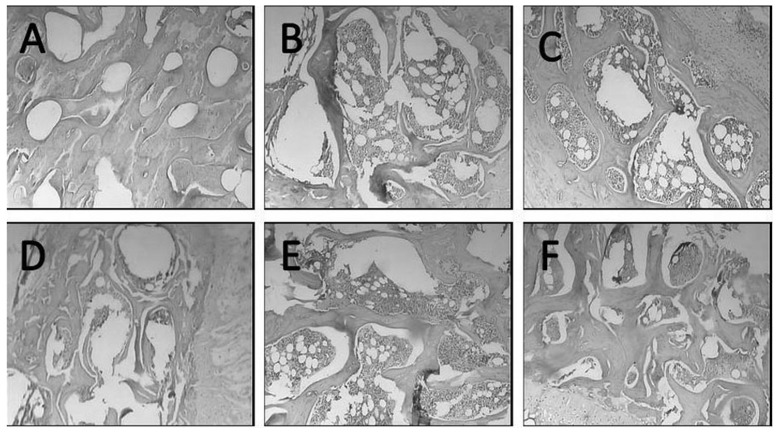
Effects of BSHY on bone tissue morphology in oophorectomized rats. (A) Control group; (B) model group; (C) nilestriol group; (D) low-dose BSHY group; (E) medium-dose BSHY group and (F) high-dose BSHY group. BSHY, *Bushen Huayu* extract.

The bone trabeculae in the model group were narrower, more lightly stained and more damaged arch-shaped connections were observed compared with the control group. Bone trabeculae fragments were also observed and the number was significantly reduced compared with the control group. In addition, the medullary cavity was enlarged in the model group and the quantity of bone marrow was increased, compared with the control group. However, compared with the model group, the bone trabeculae in the three BSHY groups and the nilestriol group were all wider, and the space and connections were markedly increased. Furthermore, the medullary cavity was reduced in size (Fig. 5).

## Discussion

Postmenopausal osteoporosis is a systematic imbalance, in which the speed of bone resorption is greater than that of bone formation. This disease is caused by estrogen deficiency and results in microarchitectural changes, particularly bone remodeling. Certain critical molecules co-ordinate the actions of osteoblasts and osteoclasts during bone remodeling (7). As a result of estrogen deficiency during bone remodeling osteoblasts release receptor activator of NF-κB ligand (RANKL), a member of the tumor necrosis factor (TNF) family. RANK binds to RANKL on osteoclasts, leading to the differentiation, proliferation, multinucleation, activation and survival of osteoclasts (8). In addition, osteoblasts release markers, including TNF-α and IL-6 (9). IL-6 generation is attenuated by sex hormones (10–12). IL-6 has been demonstrated to directly increase the viability of osteoclasts, inhibit their apoptosis and increase the length of their life cycle, resulting in osteoporosis (13,14). In the present study, BSHY-treated rats exhibited significantly elevated levels of E2, whilst levels of IL-6 were significantly downregulated, indicating that BSHY may attenuate the alterations induced in oophorectomized rats.

By contrast, ALP is a biomarker for osteoblasts and the levels of ALP have been previously demonstrated to increase when the level of estrogen increases in osteoblastic cells *in vitro* (9). However, in the present *in vivo* study, the serum levels of ALP in rats treated with BSHY from the three groups decreased (P<0.05). This may be due to the fact that half of all ALP is formed in the liver and they may cross-react in the bone ALP assay. This result is consistent with a previous study (15).

BSHY, a Chinese medicinal formulation, has been proposed to supplement the kidney system and resolve blood stasis. Among its components, Herba Epimedii (termed *Yinyanghuo* in Chinese) and the active ingredient icariin, may have a potential role in the prevention and treatment of osteoporosis by increasing the mRNA expression levels of osteoprotegerin (a TNF-related cytokine), bone morphogenetic protein (a promoter of osteogenesis) and collagen I (synthesized by active osteoblasts) in oophorectomized rats (16), as well as inducing estrogen biosynthesis (17). Rhizoma Drynariae (termed *Gusuibu* in Chinese) may enhance the treatment effects on osteoporosis by reducing metabolic disorder (18). The anti-osteoporosis mechanisms of other traditional Chinese medicines remain to be elucidated. They may have a combined synergistic effect with Herba Epimedii and Rhizoma Drynariae to enhance the actions and/or reduce the side-effects.

Further studies are required to investigate whether other bone resorption and bone formation parameters, including RANKL, RANK, tartrate-resistant acid phosphatase, IL-1, calcium, osteocalcin, TNF-α and deoxypyridinoline, are also involved in BSHY treatment in oophorectomized rats.

In conclusion, in the present study, BSHY extract was demonstrated to have a therapeutic effect on osteoporosis caused by oophorectomy, and thus may be a potential therapeutic treatment for postmenopausal osteoporosis. Furthermore, these results suggest that the mechanism by which BSHY decreases the serum levels of IL-6 may be by regulating E2.
